# Impaired Cerebral Autoregulation-A Common Neurovascular Pathway in Diabetes may Play a Critical Role in Diabetes-Related Alzheimer’s Disease

**Published:** 2017-06-05

**Authors:** Shashank Shekhar, Shaoxun Wang, Paige N Mims, Ezekiel Gonzalez-Fernandez, Chao Zhang, Xiaochen He, Catherine Y Liu, Wenshan Lv, Yangang Wang, Juebin Huang, Fan Fan

**Affiliations:** 1 Department of Neurology, University of Mississippi Medical Center, USA; 2 Institute of Clinical Medicine, University of Turku, Finland; 3 Department of Pharmacology and Toxicology, University of Mississippi Medical Center, USA; 4 Department of Endocrinology and Metabolism, The Affiliated Hospital of Qingdao University, China; 5 Department of Urology, Fudan University, China

**Keywords:** Autoregulation, Myogenic response, Diabetes, Alzheimer’s, Dementia, Rat model, T2DN

## Abstract

Alzheimer’s disease (AD) is the leading cause of progressive degenerative dementia. The hallmark pathological features include beta amyloid deposition and neurofibrillary tangles. There has been a strong association of AD with Diabetes (DM) based on human studies and animal experiments. The hallmark features of AD seem to have an exaggerated presence in AD with DM, especially type 2 diabetes (T2D). In addition, insulin resistance is a common feature in both diseases and as such AD has been called type 3 diabetes. Furthermore, impairment of cerebral autoregulation has been reported in both animal and human diabetic subjects. Cerebral vascular impairment has also been implicated in the pathophysiology of AD. There is an urgent need to develop animal models of AD and DM to explore the neuropathological mechanisms of these disease and utilize such models to develop treatment strategies.

## Introduction

Alzheimer’s disease (AD) and diabetes (DM) are two of the leading ageing related disorders. AD prevalence accounts for an estimated 5.4 million Americans in 2016 [1], where as DM affects more than 29 million Americans in 2013 [2]. AD is the only leading cause of death (6th overall) [3] that lacks any therapy to slow or reverse its progression [4] followed by DM as the 7th leading cause of death in United States (US). The Medicare cost for the treatment of dementia and AD is $159 billion annually and is projected to rise to $511 billion by 2040 [5,6]. Similarly, DM prevalence is projected to triple by 2050 which costed the nation $245 billion per year in 2012 [2]. These untreatable chronic disorders will become a major economic burden long term. Thus, there is an urgent need to understand the mechanisms of these diseases in order to develop new therapeutic strategies that delay their progression.

## Discussion

### High comorbidity of DM and AD

AD is one of the most common forms of progressive degenerative brain disorders resulting in dementia [7,8]. AD is characterized by a decline in short term memory, problem-solving, complex cognitive skills and later language dysfunction. Loss in ability to perform everyday activities requires constant nursing and long-term dependence. This decline occurs because of wide spread cortical neuronal loss in areas of brain responsible for cognitive function. Whereas, DM is a variable disorder of carbohydrate metabolism resulting in hyperglycemia, which, if persists chronically, can lead to systemic complications including cognitive impairment T2D, which begins as insulin resistance and is the most common form of DM. Numerous studies demonstrate that diabetics are at an increased risk of developing AD especially in the elderly. As a result, AD has been proposed as Type 3 DM in appropriate context [9]. Recent animal studies are proposing an increased association of T2D with AD [10,11]. This association has also been corroborated in human epidemiological studies [12,13].

A clear mechanism underlying AD has yet to be fully understood. Earlier hypotheses of neuro degeneration in AD relied heavily on cholinergic deficiency, extracellular amyloid beta (Aβ) plaque formation, and hyperphosphorylated Tau protein induced neurofibrillary tangles [14]. However, current treatments and clinical trials targeting these pathways, such as using inhibitors of acetylcholinesterase [15], and γ secretase [16–19] or immunotherapy targeting to Aβ and Tau [18], have not been proved to be able to stop or slow down the disease process of AD. Lack of effective pharmacological interventions has led the community to reconsider alternatives [14]. There is increased evidence indicating that cerebral vascular dysfunction plays an important role in the development of dementia and AD. A vascular pathogenesis has thus been proposed which comprises cerebral hypoperfusion, blood-brain barrier (BBB) dysfunction [14,20,21] and impaired cerebral microcirculation [22,23]. Diabetics with AD have increased numbers of beta amyloid plaques, tau-positive cells, advanced glycation end products and more activated microglia than the brains of AD patients without diabetes. These effects are markedly seen in the hippocampus [24]. The proposed mechanisms include insulin resistance [25], inflammation [26] and impaired glucose transporters [27]. However, there is additional impairment in cerebral autoregulation [28] resulting in microinfarction, hemorrhages, and eventual neuronal loss.

### Cerebral Autoregulation

Cerebral autoregulation was first described by Lassen in 1959, where he reported clinical studies assessing cerebral blood flow [29]. Since then, cerebral autoregulation has been broadly used to describe the local circulatory changes as well the global perfusion related changes in the brain [30]. For this review, we will use the cerebral autoregulation as blanket definition which encompasses both mechanoregulation as well chemoregulation. Perfusion related change occurring in large vessels has been described else where as mechanoregulation, where as, vascular changes occurring in response to changes in arterial CO2 is described as chemoregulation or metabolic regulation [30,31]. Furthermore, changes occurring locally around neurovascular junction are referred to as neurovascular coupling [30]. Cerebral autoregulation is an inherent mechanism where by the cerebral vasculature maintains constant cerebral blood flow by responding to systemic changes in blood pressure and thus maintaining neurovascular homeostasis [32–34]. Impaired cerebral autoregulation has been reported with advancing ageing [35–37], hypoxemia/ischemia [35] and hyperglycemia [38], suggesting these conditions are related to dysfunction at the autoregulatory pathway. Thus, it is important to understand the pathophysiology of cerebral autoregulation. The vessel’s ability to autoregulate with rise and drop in blood pressure is achieved mainly through myogenic response, and additional enhancement is achieved through metabolic activators [39]. Vascular smooth muscle cells (VSMC) are the main contractile vascular structures and are predominantly located in the wall of cerebral arteries as well pial and penetrating arterioles. These cells respond to pressure elevation by a constriction mechanism using Bayliss myogenic response [40]. Such response has been observed [33,34,41] in the middle cerebral artery territory (MCA) of the rats, where large diameter arteries (202 μm) display greater myogenic response between 60–100 mmHg, whereas penetrating arterioles (58 μm) show greater response between 20–16mmHg [42]. The myogenic response is enhanced by vasoconstrictors, e.g. Angiotensin II, ET1, and 20 HETE [33,43]. In contrast, during drop in blood pressure, vessels dilate in response to metabolic active vasodilators, e.g. Nitric Oxide (NO), endothelial derived hyperpolarizing factor, adenosine, extracellular K+, hydrogen ion, lactate, and carbon monoxide (CO) [44]. These metabolites are released at the level of neurovascular coupling from endothelial cells, and glial cells [45], including astrocytes [46], due to hypoxemia (reactive hyperemia) [47,48] or neuron activation (functional hyperemia) [45,49]. Thus, any dysfunction of these smooth muscles, endothelial and glial cells could result in autoregulatory dysfunction. Furthermore, the degree of vascular remodeling also contributes to the regulation of cerebral mechanoautoregulation. Increased vascular wall thickness and perivascular fibrosis could affect vascular compliance and decrease the ability of a blood vessel wall to expand in response to changes in blood pressure [50,51]. Enhanced vascular remodeling and decreased compliance has been reported in DM [52,53] as well in AD[54–56].

### Cerebral autoregulation, DM and AD

Aging results in impairment of autoregulation which increases the risk of cerebral pathology including stroke, vascular cognitive impairment [57–60], and AD [60–62]. The risk is increased with coexistence of hypertension and diabetes [63]. With ageing, there is increased rarefaction of small penetrating arteries to deeper structures of the brain especially the basal ganglia and periventricular white matter [59,62,64]. This results in compromised regional blood flow and formation of lacunar infarctions, as well microbleeds, all of which are correlated with decline in cognitive function [62,65,66]. As ageing advances, there is BBB breakdown, vascular remodeling, glial cell activation, and inflammation further exacerbating the neurodegeneration [51,58–60,67,68]. Evidence suggests that the myogenic response of the MCA is impaired in AD [44] and DM [69]. Persistent hyperglycemia is associated with cerebral vascular dysfunction, BBB leakage, and inflammation that may contribute to the development of neurodegeneration and eventually dementia. In AD, there is reduction in number of microvessels, VSMCs and flattening of endothelial cells [70], suggesting AD may be linked to impaired cerebral autoregulation. The Atherosclerosis Risk in Communities-Neurocognitive Study (ARIC-NCS) population, especially the diabetic population, was noted to have mild cognitive impairment (the early stage of AD) [12]. Two-hit hypothesis was first described by Zlokovic, BV. According to this hypothesis, there are vascular medicated injuries occurring from DM, Hypertension, and Stroke, which ensue a non-amyloidogenic pathway resulting in dementia [21]. In DM, arteriosclerosis occurs due to glycosylation, and as a result, vessels lose the stretch reflex, transferring the arterial pressure to the capillaries which in turn results in vascular leakage through breakdown of the BBB and oligemia (local reduction in blood flow): this last step is described as first hit [21]. consequently, the breakdown of BBB results in microinfarction, microbleeds, toxic accumulation and less clearance of Aβ proteins. Whereas, oligemia leads to APP expression and increased AB production which result in excess of Aβ: this step is described as second hit [21]. This furthers the cascade and thus perpetuates neuronal dysfunction and injury resulting in cognitive decline, and neurodegeneration [21,62,65].

Indeed, insulin resistance and glucose transporter dysfunction in the brain play important roles in T2D related AD. In a recent cohort study of about 1500 patients with T2D, researchers treated patients with Metformin vs. other hypoglycemic agents in order to observe change in cognition. They found that metformin intervention significantly reduced the risk of developing dementia by 20% when compared other diabetic therapies [71]. In another study, the use of sulfonylureas and metformin over 8 years, resulted in a decreased risk of dementia by 35% [72]. In addition, the amyloid precursor protein (APP) gene, which is associated with some cases of AD, has been shown to be involved in the insulin pathway. Therefore, impairment of this pathway can result in T2D [73]. On the other hand, impaired glucose utilization in mice via overexpression APP has been reported to cause derangement of CBF [74]. Furthermore, reduced expression of the glucose transporter GLUT1 [75,76] and GLUT3 [75,77] exacerbates AD, thus exacerbating the risk of dementia with each severe hypoglycemic episode in elderly diabetic patients [78–80].

### Ideal Animal Models for Future Studies

To further elucidate the common pathology in AD and DM, there is need for an ideal animal model. A mixed mice model of T2D and AD has been generated by crossing APP/PS1 mice (AD model) with db/db mice (T2D model) [81]. This model exhibits microglia activation, BBB leakage, brain atrophy, and tau pathology. More recently, our group used a rat T2D model-T2DN, and found that it is associated with impaired autoregulation of CBF, glial activation, inflammation and Alzheimer-like cognitive deficits [82,83]. The T2DN rats closely mimic changes in diabetic patients and develops diabetic nephropathy at 6 months of age due to impaired renal autoregulation [84–86]. Nevertheless, both animal models exhibit cerebral vascular dysfunction suggesting a greater need to explore their common ground of vascular pathology.

## Conclusion

AD and T2D are age dependent diseases. There are several potential mechanisms that have been proposed to be involved in the pathogenesis of AD including classical Aβ protein deposition, tau associated neurofibrillary tangles as well as the acetylcholine deficiency. Previous generations of treatment focusing on these mechanisms have failed to prevent the progression of AD, giving rise to the need for alternative therapeutic approaches. Recent studies have suggested that insulin resistance and cerebral autoregulation could be responsible for common pathogenesis in comorbid AD and DM. It is possible that impaired autoregulation is occurring very early before the onset of dementia. Whether this cerebral vascular dysfunction precedes neurodegeneration or whether it is simply an outcome of amyloid and tau deposition has yet to be validated. In order to identify this pathology and even to develop therapeutic interventions there is a great need for the development of an ideal animal model. The recent data on mixed T2D and AD mice and T2DN rat models are promising, however, further research is required to validate whether these models are ideal for mechanisms involved in “type 3 DM,’ especially starting from the cerebral vascular function aspect.

## Abbreviations

AD: Alzheimer’s Disease
DM: Diabetes
T2D: Type 2 diabetes
Aβ: Amyloid Beta
VSMC: Vascular Smooth Muscle Cells
CO2: Carbon Dioxide
ARIC-NCS: Atherosclerosis Risk in Communities-Neurocognitive Study
BBB: Blood Brain Barrier
APP: Amyloid Precursor Protein
MCA: Middle Cerebral Artery
